# Heart rate variability during hemodialysis is an indicator for long-term vascular access survival in uremic patients

**DOI:** 10.1371/journal.pone.0172212

**Published:** 2017-03-01

**Authors:** Ya-Ting Huang, Yu-Ming Chang, I-Ling Chen, Chuan-Lan Yang, Show-Chin Leu, Hung-Li Su, Jsun-Liang Kao, Shih-Ching Tsai, Rong-Na Jhen, Woung-Ru Tang, Chih-Chung Shiao, On behalf of SMHRG (Saint Mary’s Hospital Research Group on Geriatrics)

**Affiliations:** 1 Graduate Institute of Clinical medical sciences, Chang Gung University, Taoyuan City, Taiwan (R.O.C.); 2 Department of Nursing, Saint Mary’s hospital Luodong, Yilan, Taiwan (R.O.C.); 3 Division of Nephrology, Department of Internal Medicine, Saint Mary’s hospital Luodong, Yilan, Taiwan (R.O.C.); 4 Graduate Institute of Nursing, Chang Gung University, Taoyuan City, Taiwan (R.O.C.); 5 Saint Mary’s Medicine, Nursing and Management College, Yilan, Taiwan (R.O.C); Hospital Universitario de la Princesa, SPAIN

## Abstract

**Background:**

Vascular access (VA) is the lifeline of hemodialysis patients. Although the autonomic nervous system might be associated with VA failure (VAF), it has never been addressed in previous studies. This study aimed to evaluate the predictive values of the heart rate variability (HRV) indices for long-term VA outcomes.

**Methods:**

This retrospective study was conducted using a prospectively established cohort enrolling 175 adult chronic hemodialysis patients (100 women, mean age 65.1 ± 12.9 years) from June 2010 to August 2010. Each participant received a series of HRV measurements at enrollment. After a 60-month follow-up period, we retrospectively reviewed all events and therapeutic procedures of the VAs which existed at the enrollment and during the follow-up period.

**Results:**

During the 60-month follow-up period, 37 (26.8%) had VAF but 138 (73.2%) didn’t. The values of most HRV indices were statistically increased during hemodialysis since initiation in the non-VAF group, but not in the VAF group. Among all participants, the independent indicators for VAF included higher normalized high-frequency (nHF) activity [hazard ratio (HR) 1.04, p = 0.005], lower low-frequency/high-frequency (LF/HF) ratio (HR 0.80, p = 0.015), experience of urokinase therapy (HR 11.18, p = 0.002), percutaneous transluminal angioplasty (HR 2.88, p = 0.003) and surgical thrombectomy (HR 2.36, p = 0.035), as well as higher baseline serum creatinine (HR 1.07, p = 0.027) and potassium level (HR 1.58, p = 0.037). In subgroup analysis, a lower sympathetic activity indicated by lower LF/HF ratio was an independent indicator for VAF (HR 0.61, p = 0.03) for tunneled cuffed catheter, but conversely played a protective role against VAF (HR 1.27, p = 0.002) for arteriovenous fistula.

**Conclusions:**

HRV is a useful tool for predicting long-term VAF among hemodialysis patients.

## Introduction

Hemodialysis is the most prevalent modality of renal replacement therapy in uremic patients. [1] The vascular access (VA) is the lifeline, but also a major risk factor for bloodstream infection, hospitalization, and mortality for hemodialysis patients. [1–4] VA failure (VAF) is a main cause of failure in hemodialysis. [5] In addition, the VAF is not only a major complication which accounts for 20% to 30% of hospitalization, [6] but also a cause of the high morbidity and mortality among uremic patients receiving hemodialysis. [3, 4, 7–10] Thus VA handling is an important clinical issue in the management of chronic hemodialysis patients.

The permanent types of VAs used for hemodialysis include native arteriovenous fistula (AVF), synthetic arteriovenous graft (AVG), and tunneled cuffed catheter (TCC). [1–4] Generally speaking, the risk factors for VAF are stenosis, thrombosis, and infection. [8, 9] The critical factors contributing to the thrombosis of VA include hypotension, low intra-access pressure, and low intra-access blood flow (IABF), [5, 11] while an impaired function of autonomic nervous system (ANS) plays an important role for these situations.[12]

Heart rate variability (HRV), by presenting the variation of beat-to-beat interval of heartbeat, provides a noninvasive measurement for evaluating ANS functions. Among the frequency domain indices of HRV, very low-frequency (VLF) is thought to be influenced by the thermoregulation of vasomotor tone. Low-frequency (LF) activity and normalized LF (nLF) activity—LF divided by “total power (TP) minus VLF”—are widely recognized to reflect sympathetic influence. High-frequency (HF) activity and normalized HF (nHF) activity—normalized HF by the previously-mentioned procedure—are linked to parasympathetic nervous activity. LF/HF ratio is an index of sympathovagal balance and sympathetic nervous activities, and TP represents the sum of the frequencies. However, variance of the R-R interval values (Var) indicates parasympathetic tone or reflects all the cyclic components responsible for variability in the recording period. [13–19]

Reduced HRV is disclosed as a significant risk factor for unpleased prognoses including cardiac death, all-cause mortality, development of coronary artery disease, and diabetes in many different populations. [20–29] Some investigations have evaluated the association between HRV indices and hemodynamic status during hemodialysis, [15, 30–33] and our previous work has demonstrated HRV indices as predictors for intradialytic hypotension among chronic hemodialysis patients. [34] Since hemodynamic change would precipitate VAF because of the decreased vessel flow, [35] HRV indices may serve as reliable indicators for VAF in expressing the inadequate hemodynamic response secondary to ANS dysfunction. However, the relationship between HRV indices and VAF has never been addressed in the previous investigations. Thus we designed the current study to evaluate the predictive values of the HRV indices for long-term VA outcomes.

## Material and methods

### Ethics, consent and permissions

The study was reviewed and approved by the Institutional Review Board of Saint Mary’s Hospital Luodong (No. SMHIRB-105009), and it was carried out in accordance with the approved protocols. Informed consents were waived because there was neither breach of privacy nor possible interference with clinical decisions. And the data were analyzed anonymously.

### Study design and populations

This retrospective study was conducted using a prospectively established cohort which enrolled 175 adult patients (100 women, mean age 65.1 ± 12.9 years) receiving chronic hemodialysis with stable conditions during the period from June 2010 to August 2010. Each participant had received a series of HRV measurements at the time point before hemodialysis (HRV-0, as baseline data), and three times during hemodialysis (HRV-1, -2, and -3 at initial, middle, and late phases of the index hemodialysis session, respectively). HRVs were measured using an analyzer (SSIC, Enjoy Research Inc., Taiwan) and represented as standard frequency-domain measurements, namely, VLF (0.003 ~ 0.04 Hz), LF (0.04 ~ 0.15 Hz), HF (0.15 ~ 0.40 Hz), TP, LF/HF ratio, Var, nLF, and nHF. [18, 36] The basic characteristics including demographic information, comorbid diseases, blood tests and medications were documented from patients’ medical charts at enrollment. The details of study design, participant selection, HRV measurement, and data collection were described in our previous work. [37]

After a 60-month follow-up period following the initial HRV measurements in 2010, we retrospectively reviewed and recorded all events and therapeutic procedures of the VAs which existed at the enrollment and during the follow-up period. Then the participants were categorized into two groups (VAF and non-VAF groups) based on the outcomes of the VAs. VAF was defined as dysfunction of the VA necessitating replacement by another VA for hemodialysis use.

### Endpoint of this study

The endpoint of this study was VAF censored at 60 months. The censoring period was calculated from the date of receiving HRV measurements to the date of VAF in VAF group, or 60 months in non-VAF group.

### Statistical analysis

The Scientific Package for Social Science (PASW Statistics for Windows, Version 22.0, Chicago: SPSS Inc) and *R* 3.3.1 (R Foundation for Statistical Computing, Vienna, Austria) software were used for statistical analyses. Categorical and continuous variables were expressed as numbers (percentages) and mean [standard deviation (SD)], respectively. In all statistical analyses, two-sided p < 0.05 was considered statistically significant.

The chi-square test for categorical variables, and independent t-test for continuous variables were used to compare the data between VAF and non-VAF groups. Mixed model was applied to compare the differences among the values of the four measurements (HRV-0 to -3) of the individual HRV indices and the beta coefficients (B) of the individual HRV indices. If any significant difference in the comparison was revealed, a Post-Hoc test with Bonferroni analysis was further applied to compare the difference between any two values.

Furthermore, multivariate mixed models were undertaken to calculate the adjusted B of the individual HRV indices. In this step, all variables were put into the mixed model. The variable, which was insignificant in the model, would be deleted one after another until some significance showed in the mixed model. The first-order autoregression covariance model was used to test the influence of HRV indices. In the next step, the multivariate Cox regression method was used to determine the independent risk factors for VAF among basic characteristics and procedures. The variables put into the model contented all variables listed in Tables 1 and 2 with the exception of the repeatedly measured HRV indices.

**Table 1:** Comparisons of the basic characteristics and clinical variables between the two groups.

	VAF	Non-VAF	p-value
	(n = 37)	(n = 138)	
Gender, woman	21 (56.8)	79 (57.2)	0.957
Age, years	64.2 (11.8)	65.4 (13.2)	0.626
Period of dialysis, years	6.8 (6.2)	5.5 (5.2)	0.210
**Causes of uremia**			0.358
Diabetic nephropathy	15 (40.5)	40 (29.0)	
Hypertension	0 (0.0)	2 (1.4)	
Chronic glomerulonephritis	19 (51.4)	73 (52.9)	
Others	3 (8.1)	23 (16.7)	
**Comorbidities**			
Diabetes mellitus	16 (43.2)	49 (35.5)	0.387
Period of diabetes, years	7.5 (11.3)	4.2 (7.9)	0.100
Management of diabetes			0.713
Oral anti-diabetic drugs	6 (16.2)	27 (19.6)	
Insulin	6 (16.2)	16 (11.6)	
Hypertension	28 (75.7)	100 (72.5)	0.695
Period of hypertension, years	5.9 (8.6)	4.1 (6.4)	0.177
Taking Beta- blockers or ACEi/ARB	13 (35.1)	46 (33.3)	0.837
Hypotension	9 (24.3)	23 (16.7)	0.285
Taking midodrine	3 (8.1)	13 (9.4)	0.806
Coronary artery disease	11 (29.7)	32 (23.2)	0.412
Heart failure	7 (18.9)	36 (26.1)	0.368
Cerebrovascular disease	5 (13.5)	18 (13.0)	0.940
Peripheral arterial disease	3 (8.1)	10 (7.2)	0.859
Chronic obstructive pulmonary disease	2 (5.4)	17 (12.3)	0.230
Liver cirrhosis	2 (5.4)	14 (10.1)	0.374
Malignancy	4 (10.8)	14 (10.1)	0.906
**Types of vascular access**			0.704
TCC	4 (10.8)	20 (14.5)	
AVG	11 (29.7)	33 (23.9)	
AVF	22 (59.5)	85 (61.6)	
**Experienced therapy for vascular access**			
Urokinase	2 (5.4)	0 (0.0)	0.006
PTA	15 (40.5)	31 (22.5)	0.027
Surgical thrombectomy	8 (21.6)	13 (9.4)	0.043
**Baseline data**			
Kt/V	1.47 (0.25)	1.43 (0.24)	0.376
Urea Reduction Ratio, %	76.2 (5.8)	79.0 (61.7)	0.784
Cardio-Thoracic Ratio, %	52.9 (5.2)	51.5 (4.9)	0.697
White blood cell, x10^9^/L	6.2 (1.9)	6.3 (2.2)	0.742
Hemoglobin, g/dL	9.6 (1.2)	9.8 (1.5)	0.533
Hematocrit, %	29.8 (3.5)	30.3 (4.4)	0.586
Platelet, x10^9^/L	181.7 (60.0)	175.9 (63.9)	0.621
Blood urea nitrogen, mg/dL	79.4 (20.6)	73.5 (19.8)	0.110
Creatinine, mg/dL	11.6 (6.0)	10.3 (2.3)	0.045
Calcium, mg/dL	9.3 (0.8)	9.0 (0.7)	0.020
Phosphate, mg/dL	5.1 (1.2)	4.9 (1.8)	0.358
Albumin, g/dL	3.9 (0.3)	3.8 (0.3)	0.103
Sodium, mmol/L	137.9 (3.5)	136.7 (11.1)	0.525
Potassium, mEq/L	4.9 (0.8)	4.6 (0.8)	0.026
i-PTH, ug/L	375.9 (814.8)	261.3 (341.7)	0.408
Total cholesterol, mg/dL	157.7 (33.1)	164.5 (36.1)	0.301
Triglyceride, mg/dL	134.0 (100.3)	164.2 (138.9)	0.218
Low-density lipoprotein, mg/dL	96.3 (26.8)	98.6 (31.2)	0.675
High-density lipoprotein, mg/dL	35.1 (18.9)	35.8 (18.7)	0.828
Sugar (postprandial), mg/dL	144.9 (46.5)	149.1 (57.0)	0.681

**Notes:** Values are presented as mean (standard deviation) for continuous variables or number (%) for categorical variables unless otherwise stated. P-value was calculated using Chi-square test or independent student’s *t*-test. Baseline laboratory data were the pre-dialysis data obtained when patients receiving HRV measurement. **Abbreviations:** ACEi, angiotensin converting enzyme inhibitor; ARB, angiotensin receptor blocker; AVF, arteriovenous fistula; AVG arteriovenous graft; i-PTH, intact-parathyroid hormone; PTA, percutaneous transluminal angioplasty; TCC, tunneled cuffed catheter; VAF, vascular access failure.

**Table 2:** Comparisons of the heart rate variability indices and hemodynamic status between the two groups.

	VAF	Non-VAF	p-value
	(n = 37)	(n = 138)	
**At the index hemodialysis**			
Dry weight, kg	55.5 (10.4)	58.3 (32.8)	0.609
Actual UF, kg	2.2 (0.9)	2.2 (1.0)	0.951
%UF, %	4.0 (1.5)	4.0 (1.7)	0.994
Calcium in dialysate, mg/dL	2.90 (0.3)	2.87 (0.3)	0.629
**Before the index hemodialysis session**^+^			
VLF-0	4.96 (1.20)	4.42 (1.79)	0.185
TP-0	5.52 (1.34)	5.38 (1.91)	0.735
Var-0	5.76 (1.25)	5.56 (1.79)	0.630
nLF-0	50.71 (18.14)	38.38 (22.57)	0.020
nHF-0	25.37 (12.05)	32.35 (15.17)	0.047
LF/HF-0	0.74 (0.86)	0.08 (1.24)	0.020
**Initial phase of the index hemodialysis session**			
VLF-1	4.75 (1.36)	4.99 (1.83)	0.463
TP-1	5.50 (1.29)	5.72 (1.94)	0.509
Var-1	5.62 (1.21)	5.86 (1.79)	0.442
nLF-1	44.64 (26.55)	45.05 (21.33)	0.922
nHF-1	34.84 (19.30)	31.00 (13.60)	0.173
LF/HF-1	0.16 (1.60)	0.35 (1.09)	0.403
HR-1	74.22 (5.93)	74.09 (6.58)	0.914
SBP-1	128.54 (24.09)	129.07 (26.65)	0.914
DBP-1	73.22 (12.43)	71.86 (11.65)	0.537
**Middle phase of the index hemodialysis session**			
VLF-2	4.89 (1.41)	5.33 (1.73)	0.154
TP-2	5.61 (1.51)	6.08 (1.86)	0.165
Var-2	5.76 (1.56)	6.16 (1.75)	0.212
nLF-2	44.92 (22.52)	47.68 (21.67)	0.501
nHF-2	33.55 (16.65)	29.11 (13.26)	0.093
LF/HF-2	0.27 (1.12)	0.48 (1.07)	0.302
HR-2	75.32 (7.87)	75.09 (8.18)	0.875
SBP-2	124.55 (25.62)	128.03 (25.20)	0.459
DBP-2	69.80 (16.75)	71.41 (13.42)	0.541
**Late phases of the index HD session**^+^			
VLF-3	5.03 (1.33)	5.048 (2.09)	0.970
TP-3	5.77 (1.46)	5.85 (2.18)	0.834
Var-3	5.82 (1.31)	6.05 (1.95)	0.496
nLF-3	46.30 (25.76)	47.38 (21.97)	0.802
nHF-3	28.48 (16.41)	28.82 (12.89)	0.892
LF/HF-3	0.45 (1.22)	0.49 (1.07)	0.851
HR-3	73.84 (7.93)	76.04 (8.13)	0.142
SBP-3	123.65 (21.41)	126.27 (25.29)	0.565
DBP-3	69.60 (11.70)	71.00 (11.87)	0.522

**Notes:** Values are presented as mean (standard deviation) for continuous variables. P-value was calculated using independent student’s *t*-test. HRV-0, -1, -2, and -3 were HRV measured before hemodialysis, and at initial, middle, and late phases of the index hemodialysis session, respectively.

+ denotes with missing data.

**Units:** Ln (ms^2^) in VLF, LF, HF, TP, and Var; ln (ratio) in LF/HF ratio; normalized unit in nLF and nHF. **Abbreviations:** DBP, diastolic blood pressure; HR, heart rate; Ln, nature logarithmical; nHF, normalized high-frequency; nLF, normalized low-frequency; SBP, systolic blood pressure; TP, total power; UF, ultrafiltration; VAF, vascular access failure; Var, variance of the R-R intervals; VLF, very low-frequency.

Finally, the joint modeling method [38] was applied to determine the independent indicators among the HRV indices, which were adjusted with the independent risk factors among the basic characteristics and procedures. The joint modeling method could perform simultaneous analyses of repeated measurements and survival data, which traditionally was an impossible task.

**Fig 1:**
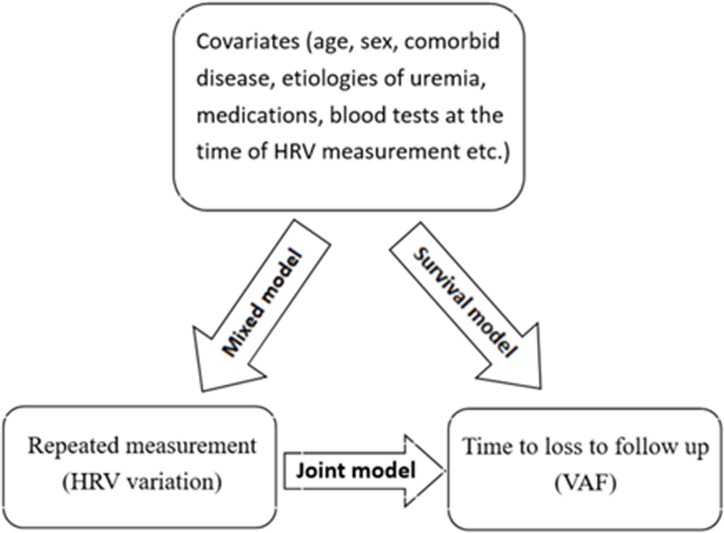
Schematic representation of the Joint modeling method. **Abbreviations:** HRV, heart rate variability; VAF, vascular access failure.

The diagrammatical representation of the joint model for repeatedly measured data and survival data had been depicted in Fig 1. The main objective of the current study was to build a joint model for modeling the repeated HRV measurements and time to the VAF process simultaneously, and to link them using unobserved random effects through the use of a shared parameter model.

## Results

Among the 175 participants, 37 (26.8%) and 138 (73.2%) were categorized into VAF and non-VAF groups, respectively, according to their VA outcomes within the 60-month follow-up period. In the VAF group, the median time from HRV measurement to VAF was 10.0 ± 7.2 months.

### Comparisons of demographic data between the two groups

The demographic data, comorbidities, and types of VAs were not of significant difference between the VAF and non-VAF groups. As for the baseline laboratory tests, only serum creatinine (11.6 ± 6.0 versus 10.3 ± 2.3 mg/dL, p = 0.045), calcium (9.3 ± 0.8 versus 9.0 ± 0.7 mEq/L, p = 0.020) and potassium (4.9 ± 0.8 versus 4.6 ± 0.8 mEq/L, p = 0.026) were significantly higher in the VAF group compared to that of the non-VAF group. The VAF group also had a significantly higher portion of receiving therapy for VA than the non-VAF group; these therapies included urokinase therapy (5.4% versus 0%, p = 0.006), percutaneous transluminal angioplasty (PTA) (40.5% versus 22.5%, p = 0.027) and surgical thrombectomy (21.6% versus 9.4%, p = 0.043). (Table 1)

### Comparisons of HRV indices during hemodialysis

**Fig 2:**
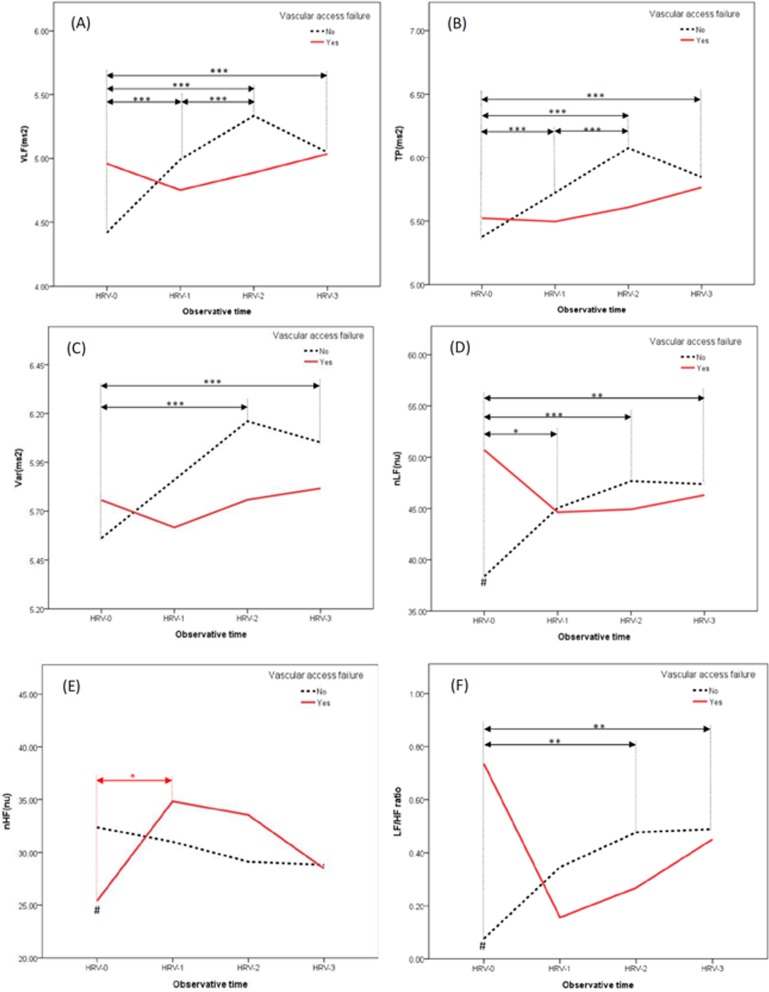
Plots comparing heart rate variability indices between the two groups. **Notes:** The indices included VLF (Fig 2A), TP (Fig 2B), Var (Fig 2C), nLF (Fig 2D), nHF (Fig 2E), and LF/HF ratio (Fig 2F). Red solid line denotes VAF group, while black dotted line denotes non-VAF group. HRV-0, -1, -2, and -3 were HRV measured at baseline, along with initial, middle, and late phases of the index hemodialysis session, respectively. *, **, *** denote p-value < 0.05, < 0.01, < 0.001, respectively, when comparing the values between two time points in the same group. Red-color denotes for VAF group, while black-color denotes for non-VAF group. **#** denotes p-value < 0.05 when comparing the values between the two groups at the HRV-0. **Abbreviations:** HRV, heart rate variability; nHF, normalized high-frequency; nLF, normalized low-frequency; TP, total power; VAF, vascular access failure; Var, variance of the R-R intervals; VLF, very low-frequency; LF/HF ratio, low-frequency/ high-frequency ratio.

Compared to the non-VAF group, those in the VAF group had higher nLF (50.71 ± 18.14 versus 38.38 ± 22.57, p = 0.020) and LF/HF ratio (0.74 ± 0.86 versus 0.08 ± 1.24, p = 0.020), but lower nHF (25.37 ± 12.05 versus 32.35 ± 15.17, p = 0.047) before hemodialysis initiation (HRV-0). Other HRV indices and hemodynamics at initial, middle, and late phases of the index hemodialysis session were not statistically different between the two groups. (Table 2 and Fig 2)

In terms of the serial change of the individual HRV indices, statistically increased values of HRV-1, -2, -3 compared to that of the HRV-0 were exhibited in most HRV indices (VLF, TP, Var, nLF, and LF/HF ratio) of the non-VAF group. In more detailed description, the values were significantly increased from HRV-0 to HRV-2, followed by a slight decrease from HRV-2 to HRV-3. In contrast, no statistical differences among the results obtained at different hemodialysis phases (from HRV-0 to HRV-3) were revealed in any individual HRV indices except the nHF in the VAF group. The value of the nHF was significantly increased from nHF-0 to nHF-1. Nonetheless, a decreasing trend of initial hemodialysis (from HRV-0 to HRV-1) followed by a subsequently increasing trend (from HRV-1 to -3) of some HRV indices (VLF, TP, Var, nLF, and LF/HF ratio), as well as an opposite trend of nHF, could be seen. (Table 3 and Fig 2)

**Table 3:** Comparisons of the levels of serial HRV measurements using mixed model.

		HRV-0^+^	HRV-1	HRV-2	HRV-3^+^	Post Hoc
	VLF	4.94 (1.79)	4.81 (1.83)	4.95 (1.73)	5.08 (2.09)	—
	TP	5.59 (1.91)	5.55 (1.94)	5.66 (1.86)	5.82 (2.18)	—
VAF group	Var	5.77 (1.79)	5.73 (1.79)	5.89 (1.75)	5.91(1.95)	—
(n = 37)	nLF	46.60 (22.57)	44.67 (21.33)	44.95 (21.67)	46.34 (21.97)	—
	nHF	27.33 (15.17)	35.04 (13.61)	33.69 (13.26)	28.59 (12.89)	(0) < (1)*
	LF/HF	0.74 (0.86)	0.16 (1.60)	0.27 (1.12)	0.45 (1.21)	—
	VLF	4.32 (1.20)	4.99 (1.36)	5.34 (1.41)	5.07 (1.33)	(0) < (1)***, (2)***, (3)*** (1) < (2)*
	TP	5.23 (1.34)	5.72 (1.29)	6.08 (1.51)	5.87 (1.46)	(0) < (1)*, (2)***, (3)* (1) < (2)*
Non-VAF group	Var	5.43 (1.25)	5.86 (1.21)	6.16 (1.56)	6.07 (1.31)	(0) < (2)***, (3)***
(n = 138)	nLF	38.76 (18.14)	44.98 (26.55)	47.60 (22.52)	47.46 (25.76)	(0) < (1)*, (2)***, (3)**
	nHF	32.25 (12.05)	31.07 (19.30)	29.19 (16.65)	28.80 (16.41)	—
	LF/HF	0.10 (1.24)	0.35 (1.09)	0.48 (1.07)	0.49 (1.07)	(0) < (2)**, (3)**

**Notes:** Values are presented as mean (standard deviation) for continuous variables. P-value was calculated using independent student’s *t*-test. HRV-0, -1, -2, and -3 were HRV measured before hemodialysis, and at initial, middle, and late phases of the index hemodialysis session, respectively. Post-Hoc analysis with Bonferroni testing was further applied if any statistical significance was found among the values at HRV-0, -1, -2, or -3.

+ denotes with missing data.

*, **, *** denote p-value < 0.05, < 0.01, < 0.001, respectively.

**Units:** Ln (ms^2^) in VLF, LF, HF, TP, and Var; ln (ratio) in LF/HF ratio; normalized unit in nLF and nHF. **Abbreviations:** HRV, heart rate variability; nHF, normalized high-frequency; nLF, normalized low-frequency; TP, total power; VAF, vascular access failure; Var, variance of the R-R intervals; VLF, very low-frequency.

### The variation of HRV measurements during hemodialysis

The variation of several HRV indices including VLF, TP, Var, nLF, and LF/HF ratio, were noticed to increase during the index hemodialysis session. By using the multivariate mixed model, we found some factors associated with the individual HRV indices. The VLF had a positive association with systolic blood pressure (B 0.01, 95% confidence interval [CI] 0.00 ~ 0.012, p = 0.027) during hemodialysis, but had a negative association with the oral anti-diabetic drug (OAD) (B -0.95, 95% CI -1.30 ~ -0.61, p < 0.001), the subcutaneous insulin therapy (B -1.36, 95% CI -1.77 ~ -0.95, p < 0.001) and the peripheral arterial disease (PAD) (B -0.96, 95% CI -1.47 ~ -0.46, p < 0.001). The TP had a negative association with the OAD (B -1.11, 95% CI -1.47 ~ -0.75, p < 0.001), the insulin (B -1.63, 95% CI -2.06 ~ -1.21, p < 0.001), and the PAD (B -0.96, 95% CI -1.48 ~ -0.43, p < 0.001), but had a positive association with the calcium concentration of dialysate (B 0.95, 95% CI 0.49 ~ 1.40, p < 0.001). The Var had a negative association with the OAD (B -1.07, 95% CI -1.40 ~ -0.74, p < 0.001), and insulin (B -1.48, 95% CI -1.88 ~ -1.08, p < 0.001). The nLF had a positive association with baseline blood urine nitrogen (BUN) level (B 0.24, 95% CI 0.16 ~ 0.32, p < 0.001), but had a negative association with age (B -0.34, 95% CI -0.48 ~ -0.21, p < 0.001). The LH/HF ratio had a negative association with age (B -0.02, 95% CI -0.02 ~ -0.01, p < 0.001) and white blood count level (B -0.08, 95% CI -0.12 ~ -0.04, p < 0.001), but had a positive association with baseline BUN level (B 0.01, 95% CI 0.01 ~ 0.02, p < 0.001).

Thus the above-mentioned factors were taken for adjustment of the HRV indices. The mean values of these HRV indices were slightly lower after adjustment with other risk factors. The baseline HRV values before and after adjustment were 4.53 and 3.66 ln (ms^2^) in the VLF; 5.41 and 4.45 ln (ms2) in the TP; 5.60 and 5.13 ln (ms2) in the Var; 40.89 and 40.32 normalized unit in nLF; 0.21 and 0.17 ln (ratio) in the LF/HF ratio. Notably, the HRV indices including the VLF, TP, Var, nLF and LF/HF ratio had positive association with the time during hemodialysis. (Table 4)

**Table 4:** Variations of HRV measurements during hemodialysis using mixed model.

	B (95% CI)	B (95% CI) after adjustment †
VLF-0	4.53 (4.20 ~ 4.87)	3.66 (3.27 ~ 4.06)
VLF-1	0.41 (-0.02 ~ 0.84)	0.44 (0.04 ~ 0.85)*
VLF-2	0.71 (0.28 ~ 1.14)***	0.76 (0.36 ~ 1.17)***
VLF-3	0.52 (0.09 ~ 0.94)*	0.55 (0.15 ~ 0.96)**
TP-0	5.41 (5.05 ~ 5.76)	4.45 (4.04 ~ 4.86)
TP-1	0.21 (-0.21 ~ 0.72)	0.33 (-0.09 ~ 0.75)
TP-2	0.57 (0.12 ~ 1.03) *	0.64 (0.22 ~ 1.05)**
TP-3	0.42 (-0.03 ~ 0.87)	0.48 (0.06 ~ 0.90)*
Var-0	5.60 (5.27 ~ 5.93)	5.13 (4.80 ~ 5.45)
Var-1	0.21 (-0.21 ~ 0.63)	0.23 (-0.17 ~ 0.62)
Var-2	0.46 (0.06 ~ 0.89)*	0.50 (0.10 ~ 0.89)*
Var-3	0.40 (-0.02 ~ 0.82)	0.42 (0.02 ~ 0.81)*
nLF-0	40.89 (36.68 ~ 45.11)	40.32 (36.31 ~ 44.34)
nLF-1	4.07 (-1.30 ~ 9.45)	4.73 (-0.39 ~ 9.85)
nLF-2	6.21 (0.84 ~ 11.58)*	6.87 (1.75 ~ 11.99)**
nLF-3	6.26 (0.88 ~ 11.65)*	7.00 (1.87 ~ 12.14)**
nHF-0	30.95 (28.24 ~ 33.66)	31.11 (28.44 ~ 33.79)
nHF-1	0.85 (-2.61 ~ 4.31)	0.66 (-2.75 ~ 4.07)
nHF-2	-0.91 (-4.37 ~ 2.55)	-1.10 (-4.51 ~ 2.31)
nHF-3	-2.20 (-5.66 ~ 1.27)	-2.42 (-5.84 ~ 1.00)
LF/HF-0	0.21 (-0.01 ~ 0.43)	0.17 (-0.04 ~ 0.37)
LF/HF-1	0.10 (-0.18 ~ 0.37)	0.15 (-0.12 ~ 0.41)
LF/HF-2	0.22 (0.05 ~ 0.50)	0.270 (0.01 ~ 0.54)*
LF/HF-3	0.27 (0.05 ~ 0.55)	0.33 (0.06 ~ 0.59*

**Note:** Multi-variate mixed model were performed for seeking any risk factors.

**†**The factors for adjustment were chosen if significant correlations existed between them and the individual HRV indices.

The VLF was adjusted with SBP during hemodialysis, OAD, insulin, and PAD. The TP was adjusted with OAD, insulin and PAD and dialysate calcium level. The Var was adjusted with OAD and insulin. The nLF was adjusted with age and baseline BUN level. The nHF was adjusted with age. The LH/HF ratio was adjusted with age, baseline BUN and white blood cell. HRV-0, -1, -2, and -3 were HRV measured before hemodialysis, and at initial, middle, and late phases of the index hemodialysis session, respectively.

*, **, *** denote p-value < 0.05, < 0.01, < 0.001, respectively, when comparing with the values at HRV-0, respectively.

**Units:** Ln (ms2) in VLF, TP, and Var; ln (ratio) in LF/HF ratio; normalized unit in nLF and nHF. **Abbreviations:** B, beta coefficient; BUN, blood urea nitrogen; CI, confidence interval; HRV, heart rate variability; Ln, nature logarithmical; nHF, normalized high-frequency; nLF, normalized low-frequency; OAD, oral anti-diabetic drug; PAD, peripheral arterial disease; SBP, systolic blood pressure; TP, total power; Var, variance of the R-R intervals; VLF, very low frequency.

### Independent predictors for VAF among basic characteristics

Table 5 showed independent indicators for VAF among basic characteristics and procedures. The factors put into the multivariate Cox proportional hazards model included all the variables listed in Table 1, namely, age, gender, period of dialysis, causes of uremia, comorbidities and drugs, types of VAs, therapeutic procedures for VAs, and baseline clinical and laboratory data.

**Table 5:** Independent predictors for VAF among basic characteristics and procedures using Cox regression method.

	B	HR (95% CI)	p-value
Urokinase ^a^	2.41	11.18 (2.42 ~ 48.43)	0.002
PTA ^a^	1.06	2.88 (1.41 ~ 5.74)	0.003
Surgical thrombectomy ^a^	0.86	2.36 (1.06 ~ 5.23)	0.035
Serum potassium ^b^^,^^c^	0.46	1.58 (1.03 ~ 2.43)	0.037
Serum creatinine ^b^^,^^c^	0.07	1.07 (1.01 ~ 1.13)	0.027

**Note:** HRV indices were not included in this analysis.

^a^ with versus without.

^b^ the data before hemodialysis.

^c^ every increment of one unit.

**Abbreviations:** B, beta coefficient; CI, confidence interval; HR, hazard ratio; VAF, vascular access failure; PTA, percutaneous transluminal angioplasty.

These independent predictors included the experience of therapies including urokinase [hazard ratio (HR) 11.17, 95% CI 2.42 ~ 48.43, p = 0.002], PTA (HR 2.88, 95% CI 1.41 ~ 5.74, p = 0.003) and surgical thrombectomy (HR 2.36, 95% CI 1.06 ~ 5.23, p = 0.035), as well as baseline serum creatinine level (HR 1.07, 95% CI 1.01 ~ 1.13, p = 0.027) and serum potassium level (HR 1.58, 95% CI 1.03 ~ 2.43, p = 0.037). (Table 5)

### Independent predictors for VAF among HRV indices

By using the joint modeling method for Cox regression method and linear mixed model, we determined the independent indicators for VAF among HRV indices under the adjustment with other risk factors listed in Table 4. We found that nHF, which was positively associated with VAF (HR 1.04, 95%CI 1.01 ~ 1.06, p = 0.005), and LF/HF ratio, which was negatively associated with VAF (HR 0.80, 95%CI 0.59 ~ 1.01, p = 0.015), were independent indicators. (Table 6)

**Table 6:** Independent predictors for VAF among HRV indices using joint modeling method for Cox regression method and linear mixed model.

HRV	All VA				TCC	AVF	AVG
indices	(n = 175)				(n = 24)	(n = 107)	(n = 44)
	B	SE	HR (95% CI)	p-value	HR (95% CI)	HR (95% CI)	HR (95% CI)
VLF ^a^	-0.01	0.02	0.99 (0.96 ~1.03)	0.687	───	───	───
TP ^a^	0.00	0.01	1.00 (0.97 ~1.02)	0.766	2.73 (2.44 ~ 3.02) ***	───	───
Var ^a^	-0.01	0.02	0.99 (0.96 ~ 1.02)	0.381	1.90 (1.75 ~ 2.06) ***	───	───
nLF ^a^	0.00	0.00	1.00 (0.99 ~ 1.00)	0.469	───	───	───
nHF ^a^	0.03	0.01	1.04 (1.01 ~ 1.06)	0.005	1.10 (1.06 ~ 1.13) ***	───	───
LF/HF ^a^	-0.22	0.11	0.80 (0.59 ~ 1.01)	0.015	0.61 (0.17 ~ 1.05) *	1.27 (1.12 ~ 1.42)**	───

**Note:** All HRV measurements had been adjusted by mixed models and joined into Cox regression method, which had been adjusted by basic characteristic. 95% CI for the log HR = loge HR ± 1.96 × SE = log (1.10) ± 1.96 × 0.1215 = −0.143 to 0.333

^a^ every increment of one unit

*, **, *** denote p-value < 0.05, < 0.01, < 0.001, respectively.

**Units:** Ln (ms^2^) in VLF, TP, and Var; ln (ratio) in LF/HF ratio; normalized unit in nLF and nHF. **Abbreviations:** AVF, arteriovenous fistula; AVG arteriovenous graft; B, beta coefficient; CI, confidence interval; HR, hazard ratio; Ln, nature logarithmical; nHF, normalized high-frequency; nLF, normalized low-frequency; TCC, tunneled cuffed catheter; TP, total power; VA, vascular access; VAF, vascular access failure; Var, variance of the R-R intervals; VLF, very low-frequency; SE, standard error.

### Independent indicators for VAF in different types of VAs

**Fig 3:**
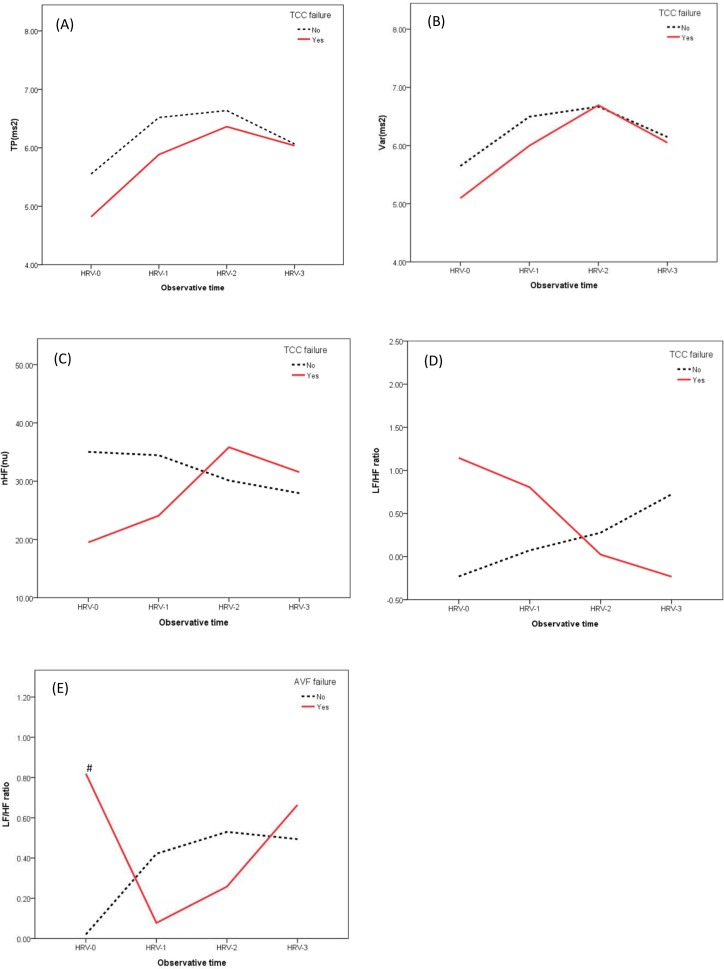
Plots comparing heart rate variability indices between the two groups in subgroup. **Notes:** The indices included TP in TCC group (Fig 3A), Var in TCC group (Fig 3B), nHF in TCC group (Fig 3C), LF/HF ratio in TCC group (Fig 3D), and LF/HF ratio in AVF group (Fig 3E). Red solid line denotes VAF group, while black dotted line denotes non-VAF group. HRV-0, -1, -2, and -3 were HRV measured at baseline, along with initial, middle, and late phases of the index hemodialysis session, respectively. No statistical difference between any two time points in the same group was found. **#** denotes p-value < 0.05 when comparing the values between the two groups at the HRV-0. **Abbreviations:** HRV, heart rate variability; nHF, normalized high-frequency; TP, total power; VAF, vascular access failure; Var, variance of the R-R intervals; LF/HF, low-frequency/ high-frequency.

In the analyses of subgroups categorized by the types of VAs, we found that TP (HR 2.73, 95% CI 2.44 ~ 3.02, p < 0.001), Var (HR 1.90, 95% CI 1.75 ~ 2.06, p < 0.001), and nHF (HR 1.10, 95% CI 1.06 ~ 1.13, p < 0.001) were positively associated with VAF, while LF/HF ratio (HR 0.61, 95% CI 0.17 ~ 1.05, p = 0.03) were negatively associated with VAF in TCC group. In the AVF group, LF/HF ratio (HR 1.27, 95% CI 1.12 ~ 1.42, p = 0.002) was the only indicator that positively associated with VAF. As for the AVG, no HRV index was disclosed as an indicator for VAF. (Table 6 and Fig 3)

## Discussions

To the best of our knowledge, the current study is the first one to address the association between ANS function, by means of HRV indices, and long-term VA outcomes. More meaningfully, we used a joint modeling method which could calculate the effects from all the repeated values, and combine both the mixed model method and survival analysis to demonstrate the predictive role for the 60-month VA survival of the repeatedly measured HRV indices. Compared to previous studies addressing the issue of HRV and hemodynamics [15, 30–33], the current method added much more value in the statistical results.

Finally, we found that higher nHF and lower LF/HF ratio, along with more experience of therapies for VA including urokinase, PTA, and surgical thrombectomy, as well as higher baseline serum creatinine and potassium levels, were independent indicators for VAF. Interestingly, lower sympathetic activity represented by lower LF/HF ratio and/or higher nHF, was an indicator of VAF for TCC but played a protective role against VAF in AVF. However, no association between HRV indices and VAF was found in AVG. (Tables 4 and 5)

### The factors associated with HRV indices

Several factors including age, impaired renal function, white blood cell count, dialysate calcium concentration, PAD, BP, DM and the therapy for DM, were identified to have influences on HRV values and thus taken for adjustment in the current study. The findings were consistent with the results of previous studies. [39–45]

### Independent indicators for VAF

The sympathetic activity increases gradually from the early stages of chronic kidney disease. [46] At an advanced stage of renal dysfunction, more than 50 percent of patients are found to have ANS dysfunction. [12] In uremic patients receiving chronic hemodialysis, a chronic sympathetic overactivation with a sympathetic withdrawal upon a more intense or prolonged sympathetic stimulation would be seen. [43, 47]

Compared to the non-VAF group, the patients with VAF had significantly higher values in nLF-0 and LF/HF-0 indicative of higher baseline sympathetic tones, and significantly lower nHF-0 value which represented lower baseline parasympathetic activity. (Fig 1)

In patients without VAF, most of the HRV indices (except nHF) which represent sympathetic or total ANS tones, tended to increase initially when the patients faced stress during hemodialysis (HRV-0 to HRV-2), but decrease a little in the later phase of hemodialysis (HRV-2 to HRV-3) when the stress increased gradually. On the contrary, this response of initial increase of ANS tone was lost in patients with VAF. (Fig 1)

In contrast to the non-VAF group, the ANS responses of the patients in the VAF group were more consistent with chronic sympathetic nervous system overactivity which was represented by higher baseline nLF and LF/HF ratio, along with a lower baseline nHF value. A subsequent sympathetic withdrawal following an increasing intense stimulation during the hemodialysis would also be witnessed. [43, 47] (Fig 1)

In the analysis from all 175 participants, lower sympathetic activity (LF/HF ratio) and higher parasympathetic activity (nHF) were independent indicators for VAF. This tendency persisted in the subgroup analysis in TCC, in which lower sympathetic activity (LF/HF ratio) along with higher parasympathetic activity (nHF) and total autonomic activity (TP and Var) were indicators for VAF. A depressed sympathetic activity, which may also appear accompanying an activated parasympathetic tone, would cause vasodilation of both arteries and veins resulting in a decreased blood pressure and blood flow in both the veins and TCC. Since TCC is a relative stiff catheter with small lumen inserted in the central veins, it is apparent that thrombosis is more likely to develop in such a situation with lower venous pressure, intra-catheter pressure, and IABF.

Nevertheless, the increased sympathetic activity conversely acted as an indicator for VAF in AVF. The thrombosis of the AVF is usually a consequence of multiple factors including stenosis, hypotension, excessive compression for hemostasis, and decreasing blood flow. [5, 11] The elevated sympathetic activity causes vasoconstriction of arteries and anastomosis site resulting in an inflow stenosis, as well as the vasoconstriction of the fistula vein, which may result in an intra-access stenosis. The stenoses of the two above-mentioned sites cause lower intra-access pressure and IABF, and subsequently bring higher risk of VA thrombosis. [48, 49] Aside from thrombosis, another important factor is endothelial dysfunction, which is also associated with elevated sympathetic activity. [50]

As for the AVG, the major etiology of thrombosis of AVG is venous outflow stenosis, [48] which is caused by intimal and fibromuscular hyperplasia in the venous outflow tract. [51] On the other hand, the relative stiff catheter with bigger lumen could not only prevent the inflow or outflow stenosis at the anastomosis sites, but also preclude the possibility of intra-access vasoconstriction or stenosis, from the effect of sympathetic activity. Thus no HRV indices were found as indicators for VAF.

Furthermore, HRV indices, urokinase therapy, PTA, surgical thrombectomy, as well as higher serum potassium levels and higher serum creatinine levels were also found as independent indicators of VAF. The experiences of urokinase therapy, PTA, and surgical thrombectomy might play both surrogate roles and causal roles for VAF. It is plausible that a VA with inadequate function would experience some therapies including urokinase therapy for TCC, as well as PTA and/or surgical thrombectomy for AVF/AVG according to the therapeutic policies or facilities of the hospital, before being replaced by another VA. At the same time, both PTA and surgical thrombectomy would cause endothelial injury, intimal hyperplasia, and atherosclerosis progression, which may attribute to the subsequent VAF. [52–55]

The higher serum potassium and creatinine levels may also play surrogate roles for VAF. In the current study, higher serum potassium and creatinine levels were correlated with higher serum blood urine nitrogen, calcium, and phosphate levels, which were further correlated with increased need of urokinase. Additionally, higher serum potassium and creatinine probably reflect a lower residual renal function and a lower dialysis clearance secondary to the dysfunction of VAs. The lower residual renal function is further associated with poor cardiovascular outcomes due to diminished clearance of middle molecular weight toxins and increased vascular calcification. The predictors of loss of residual renal function, such as diabetes, heart diseases, intradialytic hypotension, and old age, are also risk factors of VA dysfunction. [56]

### Limitations

Several limitations are worth mentioning. First, although we had excluded patients with dysrhythmia at enrollment, we didn’t exclude patients taking some anti- hypertensive agents, which may affect HRV, due to the limitation of participant numbers. However, the percentage of these drugs’ usage is similar in the two groups. (Table 1) Second, the bias of sampling could not be excluded since the HRV indices were only measured in the index session of hemodialysis. Third, the HRV measurements were taken at baseline and three times at initial, middle, and late phases in the index hemodialysis. Information of continuous ANS change is lacking. Fourth, the sympathetic tone in our patients was evaluated by an indirect method, HRV, not by some direct methods. Nonetheless, these direct methods are invasive and less practically available, and their predictive values have yet to be determined. [43] Fifth, the participant number in the TCC group is limited (n = 24). However, the statistical power is sufficient and meaningful because the HRV values were measured for four times in each participant and the joint model method could take the repeated measurements into account.

Further prospective study enrolling more participants with more frequent or continuous HRV measurements, or randomized control trials with intervention, is warranted to evaluate the association of autonomic activity and VAF, and the predictive values of HRV indices on VAF.

## Conclusions

The function of ANS is associated with VA survival. The current prospective study with a 60-month follow-up period showed that HRV measurement is a simple and useful tool to predict long-term VAF in chronic hemodialysis patients.

## Supporting information

S1 File Regular table of the data set. **Notes:** This data set was used for: (1) comparison of the basic characteristics between VAF and non-VAF groups, (2) determining the independent risk factors for VAF using multivariate Cox regression method, and (3) determining the independent indicators among the HRV indices using joint modeling method.
(CSV)

S2 File Long table of the data set. **Notes:** This data set was used for: (1) comparison of the values of the four measurements (HRV-0 to -3) of the individual HRV indices using mixed model, (2) determining the independent indicators among the HRV indices using joint modeling method.
(CSV)
