# Correction: Heart rate variability during hemodialysis is an indicator for long-term vascular access survival in uremic patients

**DOI:** 10.1371/journal.pone.0181283

**Published:** 2017-07-07

**Authors:** Ya-Ting Huang, Yu-Ming Chang, I-Ling Chen, Chuan-Lan Yang, Show-Chin Leu, Hung-Li Su, Jsun-Liang Kao, Shih-Ching Tsai, Rong-Na Jhen, Woung-Ru Tang, Chih-Chung Shiao, On behalf of SMHRG (Saint Mary’s Hospital Research Group on Geriatrics)

There is an error in affiliation 5 for the author Chih-Chung Shiao. Affiliation 5 should read: Saint Mary’s Junior College of Medicine, Nursing and Management. Yilan, Taiwan (R.O.C).
